# White matter plasticity during second language learning within and across hemispheres

**DOI:** 10.1073/pnas.2306286121

**Published:** 2024-01-04

**Authors:** Xuehu Wei, Thomas C. Gunter, Helyne Adamson, Matthias Schwendemann, Angela D. Friederici, Tomás Goucha, Alfred Anwander

**Affiliations:** ^a^Department of Neuropsychology, Max Planck Institute for Human Cognitive and Brain Sciences, Leipzig 04103, Germany

**Keywords:** second language learning, structural connectivity, neuroplasticity

## Abstract

Adult second language (L2) learning is a challenging enterprise inducing neuroplastic changes in the human brain. However, it remains unclear how the structural language connectome and its subnetworks change during adult L2 learning. The current study investigated longitudinal changes in white matter (WM) language networks in each hemisphere, as well as their interconnection, in a large group of Arabic-speaking adults who learned German intensively for 6 mo. We found a significant increase in WM-connectivity within bilateral temporal-parietal semantic and phonological subnetworks and right temporal-frontal pathways mainly in the second half of the learning period. At the same time, WM-connectivity between the two hemispheres decreased significantly. Crucially, these changes in WM-connectivity are correlated with L2 performance. The observed changes in subnetworks of the two hemispheres suggest a network reconfiguration due to lexical learning. The reduced interhemispheric connectivity may indicate a key role of the corpus callosum in L2 learning by reducing the inhibition of the language-dominant left hemisphere. Our study highlights the dynamic changes within and across hemispheres in adult language-related networks driven by L2 learning.

## Significance

The neuroplastic changes induced by learning a second language (L2) in adulthood open new perspectives for understanding brain function. The current study shows structural changes in the language network of Arabic native speakers who learned German intensively in two phases of 3 mo each. We found a marked change in the left-hemispheric lexical-semantic system and the right fronto-temporal pathway, accompanied by decreased connectivity in the corpus callosum during L2 learning, which occurred mainly in the second period of L2 acquisition. The reduced interhemispheric connectivity suggests that the inhibitory role of the corpus callosum, relevant for native language processing, is reduced in the L2 learning phase. Our findings demonstrate a clear experience-dependent structural plasticity in the human brain during L2 learning.

Cognitive functions develop in parallel with the plastic adaptation of the brain (1–6). This suggests that the gray and white matter of the brain is altered by the acquisition of new skills and thus is modulated by lifelong experiences, such as the acquired native language (7). Second language (L2) learning in adulthood is a complex task that requires the adaptation of multiple brain systems related to a wide range of novel tasks to be mastered. To date, changes associated with L2 learning were reported to extend beyond the brain regions of the native language network in the left hemisphere (8–10), with additional involvement of the right hemisphere (11, 12), as well as plasticity in the white matter connections between the two hemispheres (13, 14). How these changes in the gray and white matter might develop during L2 learning is described in a model (15) called the Dynamic Restructuring Model (DRM).

The DRM postulates three distinct phases of structural adaptation that depend on the quantity and quality of the language learning and language switching experience. In the earliest phase, L2 learning leads to changes in the gray matter areas that support the processing of the new language. Next, in the intermediate consolidation phase, the white matter pathways connecting the language processing areas show a structural modulation. Finally, in the peak efficiency phase, the model predicts further changes in brain structure, including increased frontal white matter connectivity, leading to highly efficient L2 processing and language switching performance. However, longitudinal studies of white matter change in L2 learning in large samples of adults who have achieved proficiency beyond the beginner level are still lacking. The present study aims to investigate different phases of longitudinal white matter changes within each hemisphere and across hemispheres, and to describe the brain mechanisms involved in L2 learning.

L2 learning comprises the acquisition of a new vocabulary, which includes learning novel phonemes, phonetic categories as well as word meanings, in addition to a new grammar. At the behavioral level, it has been previously reported that lexical-semantic processing of newly learned words and simple grammar is relatively easy to acquire, and native-like performance can be achieved in L2 learners (16). In contrast, it is more difficult for late L2 learners to perform real-time syntactic analysis, and they do not achieve automatic, highly proficient syntax processing until a late stage of learning (12, 16).

At the neurofunctional level, brain imaging studies have shown that low proficient L2 learners have less overlap in brain activation between first and second language processing than high proficient L2 learners (17) and recruit additional brain areas in the right hemisphere (18). These brain areas may support language proficiency by effectively handling word retrieval (11). Comparing first and second-language brain activation in the lexical-semantic domain was found to depend on the learners’ performance, but differences in the grammatical domain depend on the age of L2 acquisition (19).

Studies focusing on the neuroplasticity of the language system as a function of L2 learning (9, 10) have reported changes in the gray matter of the bilateral inferior frontal gyrus (IFG), inferior parietal lobe (IPL), and anterior and posterior temporal lobe (TL) (13, 20–23). In particular, they include cortical gray matter changes in the bilateral TL and IPL, related to phonological and lexical-semantic memory systems that are crucial for the acquisition of the new vocabulary (24–28). Additionally, since languages differ in their syntactic and morphological rules, successful L2 acquisition also depends on the brain’s adaptation to grammatical processing (29, 30). In native language processing, semantics and grammatical rules are processed in a left-lateralized network including inferior frontal and temporal-parietal regions, which are connected via dorsal and ventral white matter pathways (31). L2 acquisition during adulthood requires neural adaptations that reach beyond the classical language network, involving the right hemisphere (8–10), playing an essential role in the early learning phases when L2 processing is not yet fully automatized (24, 32).

In addition to the reported changes in gray matter, the plasticity of the white matter language pathways in L2 learning (9) has also been suggested in previous cross-sectional studies comparing bilinguals and monolinguals (20, 23, 33–35), as well as in some longitudinal language learning studies (13, 22). These studies have shown an association between L2 acquisition and local changes in white matter parameters which were located in the bilateral inferior fronto-occipital fascicle (IFOF), the superior longitudinal fascicle (SLF), the arcuate fascicle (AF), the uncinate fascicle (UF) and the corpus callosum (CC) (13, 20, 22, 23, 33) which might be related to alterations in myelination or axonal characteristics (2, 36). White matter plasticity has been reported in relation to different aspects (i.e., novel speech sounds, vocabulary, grammar, etc.) of L2 acquisition (20, 24, 37) and respective variations across the phases of language learning (9, 10, 38).

Although it is widely accepted that language processing is dominated by the left hemisphere (31, 39), increasing evidence suggests that the right hemisphere is highly involved in L2 learning (10, 40, 41), including dynamic changes in lateralization across phases of language learning (42). In addition, differences in the CC have been reported between bilingual and monolingual participants, suggesting its involvement in L2 learning (9, 14). The CC is the structural bridge that allows the interaction between the hemispheres (43, 44). However, its role in the acquisition and use of a second language remains unclear.

There are two competing theories for the general role of the CC in interhemispheric interaction. One argues for the inhibition of the activation in the other hemisphere and the other suggests excitatory mechanisms (for reviews see refs. 45 and 46). The interhemispheric inhibition theory proposes that an area can reduce the activity in homologous contralateral areas via the CC to allow for fast and automatic processing within each hemisphere leading to functional hemispheric specialization. In contrast, the excitatory theory suggests that activation in one hemisphere facilitates the activation of homolog areas, increasing information exchange between the hemispheres. It is still an open question whether, during initial L2 learning, the role of the CC is mainly excitatory or inhibitory. However, it is well established that first language (L1) processing is strongly left lateralized (47) and the CC establishes a strong inhibition from the dominant left hemisphere on the right hemisphere (48). In contrast, early phases of L2 learning involve the right hemisphere (10) possibly due to weakened inhibition in early phases of L2 learning, hence allowing the engagement of the right homologs of the language areas. Accordingly, an increase or decrease in CC connectivity could provide support for an excitatory or inhibitory mechanism. Evidence for an inhibitory mechanism of the CC in L2 learning would be provided by a decrease in CC-connectivity, resulting in a decrease in inhibition of the language-dominant left hemisphere (47) over the right hemisphere. On the other hand, an increase in CC-connectivity would suggest excitatory interhemispheric mechanisms during language learning. In this case, increased activity in the contralateral right hemisphere would be stimulated by stronger excitation originating from language activity in the left hemisphere.

Here, we provide empirical data of longitudinal white matter changes as a function of L2 learning in two successive phases. Based on the DRM, we hypothesize that L2 learning-induced changes in structural connectivity will occur mainly after an initial beginner phase of learning, during which white matter changes are expected to be limited. In a second, intermediate consolidation phase, we expect changes in the structural connectivity of the language network. This learning phase involves both semantic processing of new vocabulary and local syntactic processing based on lexical word category and semantic information. We hypothesize that these plastic changes take place primarily in the lexical-semantic system of both hemispheres and that such changes are related to the improvement in L2 performance. Furthermore, we hypothesize that L2 learning will lead to a significant change in transcallosal connectivity, supporting the role of the CC in interhemispheric communication.

To test these hypotheses, we recruited a large group of young, healthy Arabic native-speaking participants for an intensive German language course over 6 mo to reach an intermediate proficiency (B1) level. The course consisted of an initial beginner phase of 3 mo and a consolidation phase of the same duration. After 3 and 6 mo, the participants took a standardized German language test that assessed L2 comprehension and production. At the beginning of the course and after each learning period, we acquired longitudinal high-resolution diffusion MR images and computed the white matter structural connectivity network in each participant. This structural network included the intrahemispheric connections between the language processing areas in the left hemisphere, and between their right hemisphere homologs as well as the callosal connections of these cortical areas in both hemispheres. Then, we compared the network properties between the different time points to identify changes in specific pathways and subnetworks. To test all connections for longitudinal changes in connectivity in an unbiased manner without introducing strong a priori hypotheses into the analysis, we used the recently proposed network-based statistics (NBS, 49) and a mixed-effects model (50). Changes in structural connectivity were then related to improvements in language tests to demonstrate a direct functional relevance of the detected changes in the language network.

## Results

### Improvement of L2 Performance.

**Fig. 1.**
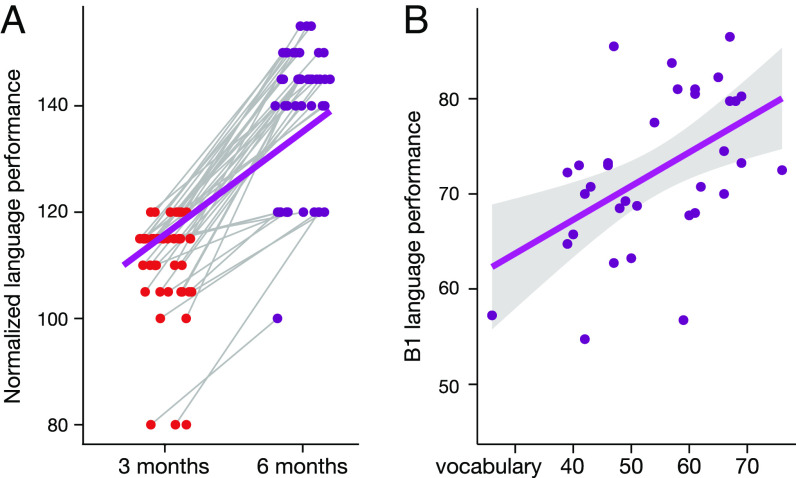
L2 improvement during learning. (*A*) Longitudinal changes of the normalized language performance from 3 to 6 mo of L2 learning. (*B*) Correlation of L2 vocabulary score and overall language performance (B1 test) after 6 mo of L2 learning.

The L2 performance after 3 mo (59 participants) and after 6 mo of learning (51 participants) was measured with standardized tests for German as an L2. The results of both tests were normalized to a common scale following the scaling method proposed in the Cambridge English Scale. Linear mixed-effects (LME) models were used in MATLAB to analyze behavioral improvement during learning, with time points modeled as a fixed effect (*Materials and Methods*). The data showed a significant improvement in L2 performance between the two time points of the German tests (t = 17.92, *P* < 0.0001, see Fig. 1*A*). After 6 mo of learning, 41 participants took an additional vocabulary test. Correlation analysis between L2 vocabulary and the B1 language test showed that individuals with richer L2 vocabulary had higher overall language proficiency (r = 0.509, *P* = 0.002, see Fig. 1*B*).

### Lateralization and Longitudinal Changes of the Intra- and Interhemispheric Connectivity.

**Fig. 2.**
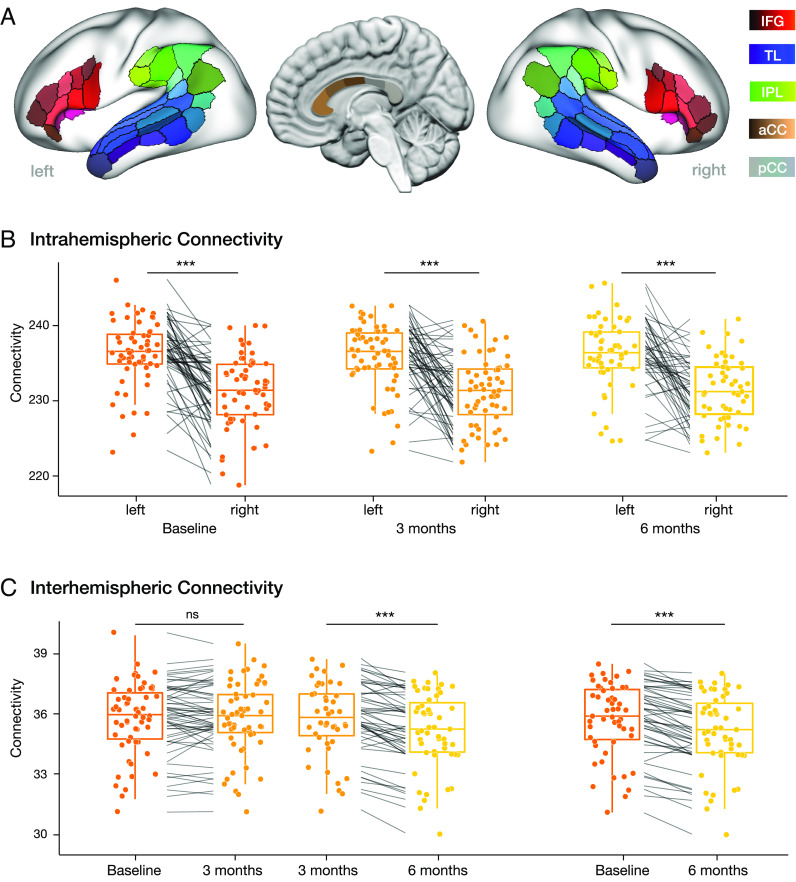
Longitudinal changes of the intra- and interhemispheric connectivity in the language network. (*A*) Areas in the language network of the left and right hemisphere in the inferior frontal gyrus (IFG), the inferior parietal lobe (IPL) the superior and middle temporal lobe (TL), and the anterior and posterior corpus callosum (aCC and pCC). (*B*) Intrahemispheric connectivity at each time point during L2 learning shows significant left lateralization of the language network. (*C*) Longitudinal changes in interhemispheric connectivity show a significant decrease in the second learning period (*Middle*) and over the full 6 mo (*Right*). The boxplots show the median, quartiles, 1.5* interquartile range, and all individual data points. (***P < 0.0001).

The initial lateralization test showed that the global intrahemispheric connectivity in the language network is stronger in the left hemisphere than in the right hemisphere for each of the three measurement time points (baseline: left > right, t = 8.17, *P* < 0.0001; 3 mo: left > right, t = 6.71, *P* < 0.0001; 6 mo: left > right, t = 6.56, *P* < 0.0001; see Fig. 2*B*). The longitudinal statistical analysis was then performed separately for the total intrahemispheric connectivity of each side and the interhemispheric connectivity using an LME model with time points as a fixed effect. The result showed a significant dynamic decrease in interhemispheric connectivity during learning, specifically with an effect in the second half of the learning period (baseline to 3 mo: t = −0.56, *P* = 0.57 (n.s.); 3 to 6 mo: t = −7.33, *P* < 0.0001, baseline to 6 mo: t = −8.97, *P* < 0.0001; see Fig. 2*C*). However, we did not observe any significant changes in the longitudinal analysis of intrahemispheric connectivity within the language network in each hemisphere or the lateralization index of the connectivity within the language network.

### Plasticity of the Structural Language Subnetworks across Different Learning Periods.

**Fig. 3.**
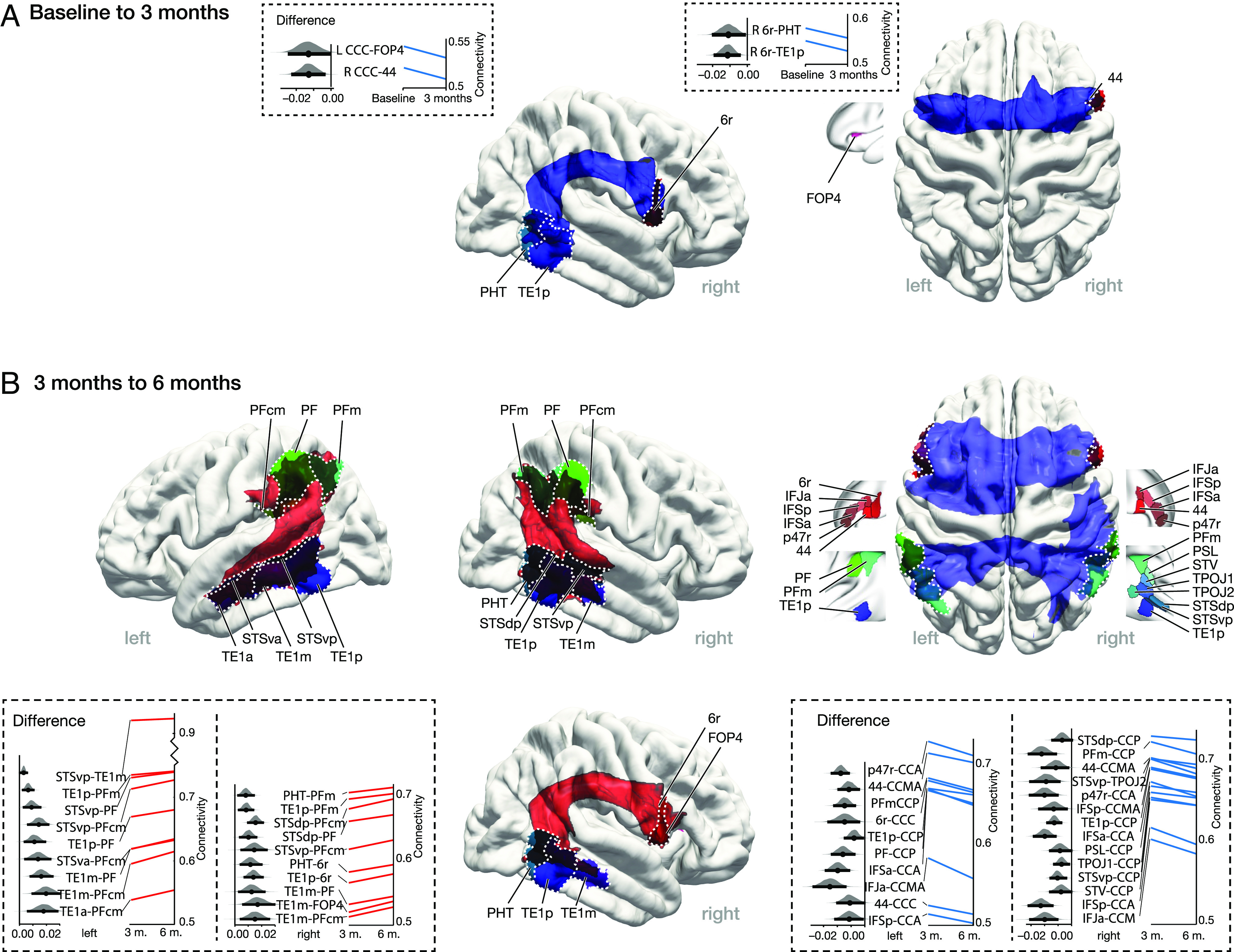
Subnetworks with longitudinally increased and decreased connectivity in the two learning periods. (*A*) First learning period: decreasing connectivity (blue) in three small subnetworks including anterior transcallosal connections as well as the right AF. (*B*) Second learning period: increasing connectivity (red) in three large subnetworks connecting the posterior temporal and the inferior parietal regions in both hemispheres along with the right AF (*Left*). Decreasing connectivity (blue) in the anterior and posterior transcallosal subnetworks (*Right*, all *P* < 0.05 NBS corrected). The brain figure shows the group averaged probabilistic tractography of the subnetworks with increased (red) and decreased (blue) connectivity together with the corresponding brain regions. The figure in the box shows the effect size and change trend of each connection.

The network-based R-statistics (NBS) LME models (p-threshold = 0.01, K = 5,000 permutations) revealed a complex reorganization of multiple subnetworks during L2 learning, including connections between all subregions in the bilateral temporal lobe (TL), inferior parietal lobe (IPL), and right inferior frontal gyrus (IFG, *P* < 0.01, NBS corrected, *SI Appendix*, Fig. S2). To shed light on the temporal properties of network changes, post hoc LME analyses between adjacent time points allowed us to identify specific effects of each connectivity for the early and later learning period (*P* < 0.05). During the first 3 mo of learning, there was a significant decrease in connectivity for only a few connections belonging to three subnetworks. These subnetworks consisted of the interhemispheric connections of subregions of the IFG and parts of the right arcuate fascicle (AF) connecting the posterior IFG and the posterior middle and inferior temporal gyrus (Fig. 3*A*). However, in the second learning period (from 3 to 6 mo), the statistical analysis revealed an increase in connectivity in three subnetworks, including the bilateral parietal-temporal system as well as the right AF (Fig. 3 *B*, *Left*). Interestingly, the frontal and temporal-parietal interhemispheric networks showed decreased connectivity in this second period (Fig. 3 *B*, *Right*). The figure shows the mean changes of all individual connections within each subnetwork and the distribution of the changes. Individual data for each participant and each connection are shown in *SI Appendix*, Fig. S6.

### Relationship between L2 Proficiency and Connectivity Changes in the Language Network.

**Fig. 4.**
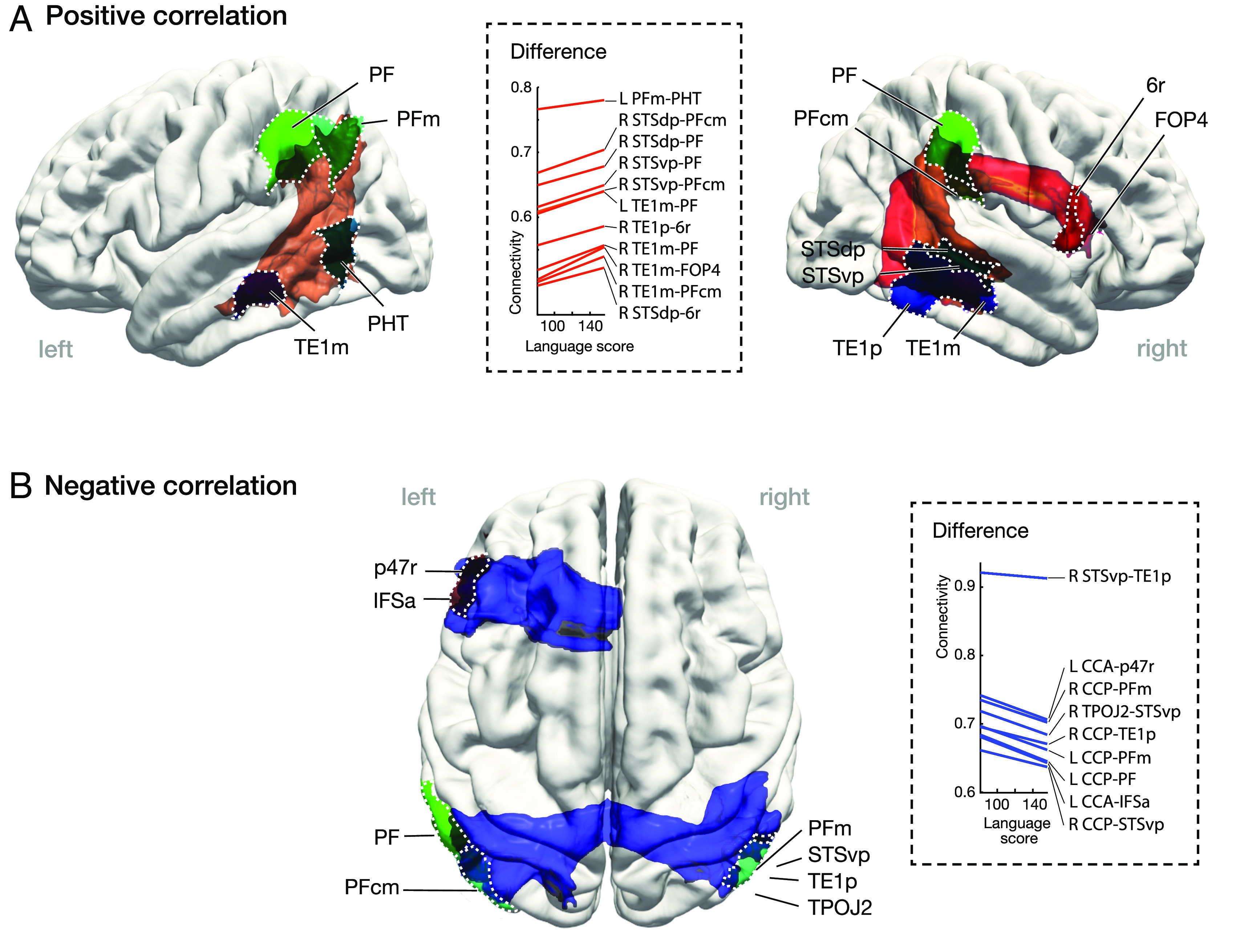
Relating changes in connectivity and the L2 proficiency from 3 to 6 mo of L2 learning. (*A*) Positive correlation between L2 performance and connectivity changes in the left and right temporal-parietal network and the AF (red). (*B*) Negative correlation in the anterior (*Left*) and posterior transcallosal network (blue, all *P* < 0.05 NBS corrected). The plots display the regression lines for all connections in all networks. In the brain images, the colored mean tractography shows the correlated subnetworks together with the corresponding brain regions. The figure in the box shows the correlation trend between each connectivity and L2 proficiency.

To test the relationship between brain network plasticity and L2 performance increase over the different learning periods, we also used NBS with LME models. The initial behavioral analysis revealed that all participants showed an improvement in their L2 scores between 3 and 6 mo of learning (Fig. 1). This monotonic increase in performance allowed us to use a more parsimonious LME model that included only the L2 score and did not require modeling time as an additional separate factor. This NBS LME model allowed us to test for longitudinal correlations between the structural network characteristics and L2 performance for each participant after 3 and 6 mo of learning. Fig. 4 shows the brain subnetworks that show a significant linear relationship between the L2 score and the brain connectivity at 3 and 6 mo (*P* < 0.01, NBS corrected). The NBS LME analysis showed that the improvement in L2 proficiency was correlated with increased connectivity in subnetworks connecting the posterior temporal and the inferior parietal lobes in both hemispheres as well as in the right arcuate fascicle (AF) (Fig. 4*A*). Additionally, a negative correlation between connectivity changes and the L2 score was found in the anterior and posterior interhemispheric connections (*P* < 0.01, NBS corrected, Fig. 4*B*). In the frontal lobe, only the left subnetwork of the transcallosal connections showed a significant correlation. The figure shows the regression lines of the correlation for all individual connections within each subnetwork. The individual data for each participant and each connection are shown in *SI Appendix*, Fig. S7.

Another measure of L2 learning success relates to L2 vocabulary size. In a post hoc analysis, we examined whether participants with a large L2 vocabulary showed different changes in white matter connectivity compared to those with a smaller L2 vocabulary. The group was divided based on their productive vocabulary in the written text of the B1 test. This vocabulary measure was chosen as it was available for all participants. It correlated strongly with B1 language performance (r =0.465, *P* < 0.01, *SI Appendix*, Fig. S3). After splitting into two groups, only the group (19 participants) with vocabulary scores above the mean showed a positive correlation between increased L2 scores and connectivity changes in the right temporal-parietal and AF subnetwork and a negative correlation with interhemispheric connectivity (*P* < 0.01, NBS corrected, *SI Appendix*, Figs. S4 and S5). This suggests an important role of the right hemisphere for successful L2 learning. However, the small sample size after splitting the groups could also explain the lack of a significant effect in the lower vocabulary group (21 participants).

## Discussion

The present longitudinal study tested the hypothesis that second language (L2) learning induces a dynamic reorganization of the structural white matter language network. For this purpose, Arabic native speakers participated in a 6-mo intensive language learning program in which they learned German as their L2. In the first half of the learning period (0 to 3 mo), only small subnetworks with a few white matter connections showed significant changes in connectivity, whereas, in the second half of the learning period (3 to 6 mo), significant changes were observed in multiple and larger white matter subnetworks. Specifically, the bilateral temporal-parietal system and the right arcuate fascicle (AF) showed increased connectivity. Additionally, the interhemispheric connectivity across the corpus callosum (CC) was reduced during this learning period. Most importantly, the brain changes in the late learning period correlated with the increases in L2 proficiency.

Our findings provide empirical evidence for the time course and location of white matter changes in the consolidation phase as suggested by the dynamic restructuring model (DRM) of second language acquisition (15). This model suggests that plasticity in the white matter language network will emerge in a second phase of L2 acquisition, allowing for more efficient interaction between the different language areas within each hemisphere. This is indeed what we found in the present study.

### Dynamic Intrahemispheric White Matter Changes Underlying L2 Performance.

The white matter network is the structural basis for neuronal communication between brain areas and its plasticity is crucial for learning new skills (2). Distinct subnetworks within the language system are specialized for different domains, and the corresponding connections are modulated by their usage. Therefore, we expected changes in the subnetworks reflecting specific tasks to be mastered in the newly learned language.

The analysis of the language test showed a significant improvement in L2 from 3 to 6 mo. L2 performance at 6 mo correlated with the results of an independent L2 vocabulary test (51). From a neuroplasticity perspective, it is important to note that we found a significant change in the structural connectivity over this period in bilateral temporal-parietal subnetworks and right temporal-frontal connections. Crucially, these longitudinal changes in connectivity were found in subnetworks that were very similar to those that showed changes correlated with L2 proficiency. The identified subnetworks form the structural basis for lexical-semantic and phonological processing (31, 52, 53) and have previously been related to L2 vocabulary learning (24, 25, 54). In this regard, it is interesting to look at subgroups of participants with higher and lower L2 vocabulary scores. Our additional correlational analysis in these subgroups revealed a significant positive correlation between L2 improvement and changes in connectivity in the right frontotemporal subnetwork as part of the AF in the second learning phase only in the group with a larger L2 lexicon (*SI Appendix*, Fig. S4). This result suggests that during this phase of L2 learning, the changes in the language network are related to the consolidation of lexical processing and highlights the importance of the right hemisphere for L2 acquisition.

Successful language learning depends on phonological discrimination during perception and phonological selection during production to decode speech sounds and associate them with the meaning of new words. In the neural language network, the bilateral inferior parietal lobe (IPL) and the superior temporal gyrus (STG) are involved in phonological storage and word decoding, and the middle temporal gyrus (MTG) is an integral region engaging in lexical-semantic access (31, 53, 55). Functional imaging studies of L2 learning (30) suggest a stronger functional activity and connectivity of the right IPL and STG during early learning phases, and phonological processing of L2 words (56, 57). Structural imaging studies demonstrate that these regions show changes in gray matter morphology during L2 learning related to L2 vocabulary acquisition and L2 proficiency (25, 26, 54, 56–59).

In addition to white matter effects in temporal-parietal connections, we also found increased connectivity in the right hemispheric temporal-frontal subnetwork as part of the AF which corresponds to the right hemispheric equivalent of the dorsal language network. This finding is consistent with previous studies highlighting the importance of the right hemisphere for L2 lexical-semantic and phonological processing during the initial and intermediate phases of adult L2 acquisition (10, 24). In addition, right prefrontal-parietal/temporal connections have been found to change in relation to L2 learning in an immersive context (23) and to be related to L2 vocabulary competence (24). Finally, a higher involvement of the right prefrontal cortex (60) during L2 processing may be related to more working memory and attentional processes in L2 (61). The white matter changes overlap with previous findings in L2 learners and experienced bilinguals, showing effects in the temporal lobe (posterior IFOF) and bilateral SLF (13, 20, 22, 23). Taken together, our findings strongly suggest that efficient L2 learning, especially vocabulary acquisition in adults, involves the right fronto-parieto-temporal network.

### Longitudinal Decrease in Transcallosal Interhemispheric Connectivity.

Comparing the network strength in both hemispheres showed that the language network is lateralized to the left at all measured time points during L2 learning. This is in line with the widely accepted model that the language network is dominated by the left hemisphere (31). In previous studies (10, 32), and the present data, there is evidence of increased right hemisphere involvement during L2 learning, as reflected by strong changes in white matter connectivity in the right hemisphere. These changes might be directly related to the changed transcallosal connectivity, allowing for additional L2 processing to occur in the right hemisphere (45). Indeed, we found a significant decrease in the interhemispheric connectivity in the anterior and posterior CC during L2 learning. This reduction correlated with the increase in L2 performance in the second learning phase. This finding confirms the previously reported central role of the CC in L2 acquisition (23, 34, 35). In particular, the microstructure of the CC shows effects of L2 experience (34, 35). Interestingly, these studies show different relationships between the age of onset of L2 acquisition and the white matter properties. The different effects could be related to different learning conditions (immersion in the L2 environment vs. classroom only in the home country) or different stages of learning, as suggested by the DRM (15). The reduction in interhemispheric connectivity at an intermediate L2 learning stage in this study complements these findings and provides a comprehensive demonstration of the role of the CC in L2 learning. In native language processing, the dominant left hemisphere exerts an inhibitory influence on the non-dominant right hemisphere via the CC (46). However, during the initial and intermediate phases of L2 learning, a highly involved right hemisphere language network is required to build up the L2 lexicon. This would explain why successful L2 acquisition is accompanied by a decrease in transcallosal connectivity. This reduces the inhibition of the language-dominant left hemisphere on the corresponding regions in the right hemisphere, allowing increased processing and connectivity to occur in the right half of the brain. However, this fundamentally new finding needs to be further explored and supported by additional data.

### Between-Subject Variability.

Participants in the study showed a considerable amount of between-subject variability in their language scores, their learning progress, and connectivity changes in brain networks. Previously, the learning differences were suggested to be related to individual differences in cognitive ability and experience (9, 10). The linear mixed-effects model allowed the incorporation of this variability into the statistical approach. However, after the same period of learning, some participants might be at a more advanced L2 level, which could relate to different stages of learning in the Dynamic Restructuring Model (DRM). Therefore, a more complex non-linear model might be helpful in future studies. The observed variability could be related to baseline differences in structural (62, 63) and functional connectivity (64). In particular, it was shown (62) that frontoparietal anatomical connectivity predicted individual L2 learning success in white matter tracts that overlapped with the language networks that changed during L2 learning in the current study. Future studies could combine a predictive and plasticity analysis including additional individual parameters to provide further insights into the variability of L2 learning.

In addition, a correlation between L2 scores and baseline interhemispheric functional connectivity was shown (64), highlighting the central role of the corpus callosum in L2 learning. The higher functional connectivity in good learners, inferred from a stronger correlation of fMRI signal between IFG areas in both hemispheres, may be related to the reported decrease in connectivity of the corpus callosum during L2 learning.

An additional source of variance is the diglossic state of the participants in this study, which may affect cognitive processes (65). In addition to the spoken Levantine Arabic, all participants learned and used Modern Standard Arabic for written and formal communication. Previously, it was shown that learning a third language appears to show similar structural changes in the white matter pathways of the brain as the acquisition of this language as a second language (66). Therefore, we don’t expect a relevant influence of the diglossic state of the participants on the presented results.

## Conclusions

Our study showed that L2 learning in adults leads to dynamic changes in brain connectivity within and across hemispheres. The experimental evidence suggests that plastic changes in the white matter system occur mainly after an initial period of learning. In this phase, the adaptation of the language network appears to be focused on the lexical-semantic system, particularly in the temporal and temporal-parietal regions, with increased connectivity in each of the two hemispheres and strong involvement of the right side. At the same time, L2 learning leads to reduced connectivity between the hemispheres, which could result in reduced inhibition of the dominant left hemisphere on the right, temporarily freeing up resources in the right half of the brain for efficient learning of new L2 linguistic features.

## Materials and Methods

### Participants.

Eighty-four young, healthy right-handed Arabic native speakers were recruited for an intensive German course (5 h/d, 5 d/wk) over 6 mo to reach the threshold level B1 according to the Common European Framework of Reference for Languages (CEFR, 67). The course was divided into two learning phases of 3 mo each. During the two learning periods, some participants left the language course for personal reasons and were not included in the corresponding analysis. Fifty-nine participants completed the first learning phase (mean age, 24.4 ± 4.5 (SD) y, 51 male) and 51 participants completed the two learning phases (mean age, 24.7 ± 4.6 (SD) y, 43 male). After each learning phase, participants took a 90-min standardized second language proficiency test (A1 and B1 tests of the Goethe Institute). After 6 mo of learning, an additional L2 Vocabulary Size Test (VST) was taken by a subgroup of 41 participants (35 male). All participants were immersed in the second language environment and lived in Germany during the course. All participants were native speakers of the Levantine dialect of Arabic and of normal intelligence [non-verbal Raven’s matrix test (68), score 50.4 ± 6.7, ranging around the upper 90 percentile of the reference population, subgroup N = 32] and spoke only one native language. They also learned and used Modern Standard Arabic in formal education and communication, making them diglossic. All participants were recruited in Leipzig and arrived in Germany 6 to 8 mo before the start of the study. When recruited, they were settled in Leipzig for a long-term stay and were highly motivated to learn German and integrate into the academic system. Before the study and after arriving in Germany, the participants had lived in refugee camps for several months. This hindered strongly their ability to learn the language and interact with the German community. However, during the first 6 mo, a stable and permanent housing situation was provided, and they enrolled in the language course offered in the study. An initial German language proficiency test revealed that all participants in the group had little-to-no knowledge of German, scoring well below the minimum for the A1 beginner level. This initial test was not adapted to capture variability at this low L2 level and was not used in the analysis. To rule out undiagnosed impairments resulting from causes related to migration to Germany, all participants were screened for symptoms of post-traumatic stress disorder (PTSD). Only those who showed no clear symptoms of mental health problems or PTSD were selected for our study. Structural MRI and high-angular and spatial resolution diffusion MRI data were acquired from each participant on a Siemens 3 T Prisma MRI scanner at baseline and after 3 and 6 mo of learning. Details of the learning procedure and MRI acquisition can be found in *SI Appendix*. The experiment was approved by the ethics committee of the University of Leipzig, and all participants gave written informed consent in their native language.

### Structural Language Connectome.

We used probabilistic diffusion MRI tractography to compute the white matter network between the language processing regions (Fig. 2*A*) in each participant and time point (baseline, 3 and 6 mo of learning). The analysis followed the previously established method (7). Cortical seed and target areas were defined using the Human Connectome Project (HCP) fine-grained atlas in addition to a subdivision of the corpus callosum (CC) atlas (69, 70). The core regions of the language network, as defined previously (31), included the dorsal and ventral pathways in both hemispheres between subregions in the bilateral inferior frontal gyrus (IFG), superior temporal gyrus (STG), middle temporal gyrus (MTG), and inferior parietal lobe (IPL). To account for interhemispheric connections, we included white matter regions in the medial cross-section of the CC resulting in 33 cortical and 5 CC regions per hemisphere (Fig. 2*A* and *SI Appendix*, Table S2). The CC is a bottleneck for estimating interhemispheric connections, and direct one-to-one connectivity between cortical areas in both hemispheres cannot be robustly estimated by tractography. Therefore, we computed probabilistic tractography between cortical and CC regions as a robust approximation of the interhemispheric connectivity. To remove false-positive connections (71), we retained the 30% strongest connections for the network analysis. Details of the connectivity analysis can be found in *SI Appendix*.

### Statistical Analyses.

To estimate longitudinal changes in the behavioral learning progress, a linear mixed-effects model (LME) (i.e., y ~ time + (1 | participant); y represents L2 proficiency) with time points as the fixed effect and participant as the random effect was applied to the scaled language test scores obtained after 3 and 6 mo of learning to analyze changes across learning periods. In addition, a correlation analysis was performed between the L2 vocabulary level after 6 mo and the B1 language test scores to analyze how strongly the composed language test is related to vocabulary knowledge at this stage in this group.

To assess the relationship between L2 learning and plasticity in the white matter language network, we first tested the changes in the overall network strength within and between brain hemispheres. We first measured the network strength in each hemisphere (sum of all connectivity values between the cortical regions) and compared this parameter between hemispheres at each time point (baseline, 3, and 6 mo of learning) to test for lateralization of the language network using a paired *t* test. Next, we used a separate LME model [i.e., y ~ time + (1 | participant); y represents connectivity] with the three measurement time points as a fixed effect to test longitudinal learning-induced changes in interhemispheric connectivity as well as intrahemispheric changes in the language network within each hemisphere (left and right) and the lateralization index. The interhemispheric network strength is the sum of the weighted connections between all cortical language regions and the CC, representing the connections crossing to the other hemisphere. In all analysis steps discussed so far, the LME models used time as a fixed effect and participant as a random effect, and the statistical tests were performed in MATLAB.

To localize subnetworks showing longitudinal changes within the language network across all time points, the recently proposed network-based R-statistics (NBS, 49) for LME models (50) with time points as a fixed effect and participant as a random effect was used. In the first step, the connectivity measures at different time points were modeled as fixed effects and participants were entered as random effects. This allowed us to analyze the longitudinal change of the structural connectome across the three different time points and account for individual differences (i.e., y ~ time + (1 | participant); y represents connectivity). Then, post hoc statistics were used to identify subnetworks with significant changes between each pair of measurement points and to determine in which of the two language learning phases white matter changes occurred. Finally, to test whether changes in connectivity were related to individual L2 performance and to localize such subnetworks, we employed a second type of model and included L2 proficiency test scores as a fixed effect and participants as a random effect in the NBS LME models, allowing us to account for the longitudinal interactions between brain structural connectivity and L2 proficiency [i.e., y ~ score + (1 | participant); y represents connectivity]. Since test scores could only be acquired after 3 and 6 mo of learning, these two time points were considered in this analysis. To visualize the statistically identified subnetworks, additionally, probabilistic tractography was computed between the regions belonging to these subnetworks. The individual pathway maps were normalized, averaged, and visualized together with the regions of the subnetwork. This allowed us to show the white matter pathways belonging to the identified subnetworks.

## Supplementary Material

Appendix 01 (PDF)

Dataset S01 (CSV)

Dataset S02 (CSV)

Dataset S03 (CSV)

## Data Availability

All connectivity measures are included in the *SI Appendix*.
