# Supplementary material for: White matter plasticity during second language learning within and across hemispheres
Source: Proc Natl Acad Sci U S A. 2024 Jan 4;121(2):e2306286121. doi: 10.1073/pnas.2306286121 (PMC10786292; doi:10.1073/pnas.2306286121)
Supplement: Supplementary file 1 — Appendix 01 (PDF) [file pnas.2306286121.sapp.pdf]

Supplementary Materials for

White matter plasticity during second language learning within and across hemispheres

Xuehu Wei, Thomas C. Gunter, Helyne Adamson, Matthias Schwendemann, Angela D. Friederici, Tomás Goucha, and Alfred Anwander

xuehuwei@cbs.mpg.de

This file includes:

Supplementary Methods

Supplementary Figures S1 to S7

Supplementary Tables S1 to S2

Supplementary Methods

Language learning procedure. In our study, we recruited a large group of young, healthy, native Arabic speakers to participate in a six-month intensive German language course to reach an intermediate level of proficiency (B1, first level of independent language proficiency). The second language (L2) teaching and assessment structure followed the Common European Framework of Reference for Languages (1, 2). According to the CEFR, the first intermediate level of independent language use (B1) can be reached after approximately 600 hours of language learning, which corresponds to a six-month intensive course. The courses have been structured in cooperation with the Herder-Institute of the University of Leipzig, Germany, which is specialized in research and teaching of German as an L2. The proficiency levels of this standard framework comprise six levels. Levels A1 and A2 represent the elementary use of the language for beginners. Levels B1 and B2 represent intermediate language levels. At the first intermediate level (B1) of German, a learner can understand the main points when clear, standard language is used and familiar topics related to work, school, leisure time, etc. are the focus. The learner can make a short statement to explain his views and plans. C1 and C2 are the highest possible levels. In our study, the participants underwent two phases (0-3 months and 3-6 months) of daily intensive classroom training in German (L2). The course combined classroom teaching using standard textbooks, complex naturalistic speaking and reading, and clear instruction in grammar and vocabulary. The course took place at the Max Planck Institute in Leipzig, in small groups of 12-15 students, 45 minutes per lesson, 5 lessons per day, 5 days per week. Three different professional teachers taught the classes in each group to increase

the language input of the learners and to reduce instructional variance between the groups. Daily homework was assigned by the teachers and consisted mainly of consolidating and reviewing the topics taught. In addition, several Arabic-speaking student assistants helped the learners with everyday issues so that they could concentrate fully on the language courses.

Language proficiency test. After 3 months and 6 months of learning, participants took a 90-minute standardized second language proficiency test that assessed language comprehension and production performance through four subtests in listening, reading, writing, and speaking in German. Language acquisition was assessed in the first phase between 0 and 3 months with the standardized A1 test and in the second phase between 3 and 6 months with the B1 language test of the German Goethe Institute, which tests oral and written, receptive and productive skills (i.e. listening, reading, speaking, and writing). This test has several advantages over a series of detailed tests of specific linguistic aspects, including motivation, comparability, focus on actual skills, practicality, and relevance to participants' lives. The total score is calculated as the sum of the 4 subtests and is therefore more robust and sensitive than the individual subtests. An additional L2 Vocabulary Size Test (VST) was taken by a subgroup of 41 participants (35 males) after 6 months of learning (3). This receptive VST was developed at the Institute for Test Research and Test Development, Leipzig (ITT-Leipzig, http://www.itt-leipzig.de, 4). The students were tested on their knowledge of a sample of the 3000 most common words required at this level. It measures in five sections, how many of a sample of words belonging to a given frequency range are known (1000 most frequent, 2000 most frequent, etc.). This results in a maximum score of 30 points per section, which were added together.

Transformation of the A1 and B1 language scores to a common scale. To estimate the L2 proficiency longitudinally and correlate it with the brain structural plasticity during learning, scores from each language test at each time point were scaled to a common scale following the Cambridge English Scale (https://www.cambridgeenglish.org/exams-and-tests/cambridge- english-scale). In our study, the progress scale was always divided into steps of 5. The detailed conversion relationship between the test score and the common scale is shown in Supplementary Table S1.

MRI data acquisition. Structural and high-resolution diffusion-weighted MR images were acquired on a 3 Tesla Prisma MRI system (Siemens Healthineers, Erlangen, Germany) with a 32-channel head coil with the following scanning parameters: Isotropic voxels resolution of 1.3 mm, 60 diffusion directions (b = 1000 s/mm^2) and 7 images without diffusion weighting (b = 0 s/mm^2), TE = 75 ms, TR

= 6 s, GRAPPA = 2, CMRR-SMS=2, 3 repetitions to improve the signal-to-noise ratio, and 2 b0 acquisitions with opposite phase encoding. The diffusion sequence was repeated 3 times to increase the. For the anatomical segmentation, we acquired quantitative multiparametric structural images with 1 mm resolution (5). The images were preprocessed using the publicly available hMRI toolbox (http://hmri.info) and the quantitative magnetization transfer (MT) images were used for the segmentation and parcellation steps.

Connectivity analysis:

Diffusion MRI preprocessing. Preprocessing of diffusion data was performed using the FMRIB Software Library (FSL, http://www.fmrib.ox.ac.uk/fsl). Diffusion images were corrected for susceptibility and eddy current induced distortions as well as head motion with the FSL tools “topup” and “eddy” using optimized parameters matched to image resolution. Imaging noise in the high-quality diffusion MRI data was minimized by combining the three repetitions. No additional denoising algorithms were applied to minimize image blurring. The optimized imaging settings allowed Gibbs ringing artifacts to be minimized and an additional correction was not required. No additional intensity or bias field correction was applied. Finally, the brain volume was masked from the background and the standard DTI contrast maps were computed. The processed datasets were checked individually to exclude artifacts from the acquisition or preprocessing. Finally, the voxel-wise fiber distribution for probabilistic tractography was computed with up to 3 fiber directions per voxel using the FSL command “bedpostX” (6).

Surface segmentation. For each participant, the structural connectome of each hemisphere was computed as follows (see also Supplementary Figure S1). First, the cortical and white matter surface, as well as the 5 subsections of the corpus callosum of each participant were generated from the MT images using FreeSurfer 5.3 (http://surfer.nmr.mgh.harvard.edu, 7). The white matter surface was shifted 1 mm into the white matter using the FreeSurfer command “mris_expand” to define robust seed and target regions for probabilistic tractography.

Parcellation of the seed regions. The cortical surface was divided into 180 regions in each hemisphere using the multi-modal parcellation developed as part of the Human Connectome Project (HCP, 8). Therefore, the atlas annotations were transformed into separate labels using “mri_annotation2label“, and then mapped to each participant using “mri_label2label”. Finally, the labeled white matter surface (shifted 1mm inside the white matter) was mapped to the individual anatomical voxel space using “mri_label2vol” to generate a voxel-based definition of parcellation

corresponding to the cortical areas. The labeled cortical and CC regions were registered to the diffusion images (FA contrast) using a rigid body registration using "flirt" and applied to the labeled regions using nearest-neighbor interpolation. Using the corpus callosum sections as seed and target regions in probabilistic tractography reduces the problem of spurious white matter connections resulting from the tracking through the bottleneck of the corpus callosum. This results in a more robust estimation of the inter-hemispheric connectivity.

Connectivity estimation. All registered regions were used as seed areas for probabilistic tractography (6) using "probtrackX" with default parameters. The structural connectivity between all regions was computed representing the relative number of streamlines between all pairs of regions. Those connectivity estimates are influenced by the local microstructural properties of the pathway between the regions and integrate the local properties into one connectivity estimate for a specific connection. The estimated connectivity values for all regions (full HCP atlas) were logarithmically scaled and normalized by the size of the seed region (log of the number of seeded streamlines) to build a connectivity matrix with normalized values ranging from zero to one. Next, the values for both tracking directions were averaged (from region A to region B, and from region B to region A). For each participant and each hemisphere, we obtained the weighted symmetric connectome matrix (Supplementary Figure S1).

Network thresholding. Additionally, we removed weak and noisy connections below a predefined threshold (in the average matrix across all participants) as they cannot be estimated reliably with tractography due to the limited sampling of the distribution that may result in false-positive connections (9). This allowed the exclusion of connections that did not align with the major fiber pathways in the human brain (10), and removed, e.g., connections between the left parietal lobe and frontal CC regions that do not exist anatomically. To determine this threshold, we increased the threshold in increments of 10% to create seven networks with different densities. Network thresholding methods were shown to be able to disentangle spurious and genuine connections (9). These networks ranged from a dense network that contained 80% of all connections to a sparse network that included only the strongest 20% per hemisphere. A threshold of 30% was found to reliably remove implausible false-positive connections and still retain the major pathways for the network-based analysis. With this global density threshold, 67% of the connections within the cortical language network (out of 528 per hemisphere, 33*32/2) and 55% of all possible connections including the CC areas were retained. The same network mask was applied to every

individual participant. Finally, the 33 language ROIs in each hemisphere were selected and the matrix was reduced to those elements for further analysis.

Network-based statistics. We used network-based statistics (NBS) (11) to identify subnetworks with systematic structural changes. NBS is a method to control for the family-wise error rate when testing each connection in the network by using the extent to which the edges are connected. Therefore, all connected components that were present in the set of supra-threshold connections (T-threshold = 3.3) were identified and the number of connections was stored. To estimate the significance of each component, NBS performed a nonparametric permutation test (K = 5000 permutations). At each permutation, the group to which each participant belonged was randomly exchanged, the same threshold was applied to create the set of connections above the threshold for each K permutation, and then the statistical test was recalculated and the size of the largest component m in the set of supra-threshold connections was stored. The p-value of each connected component of size m was then estimated by searching for the proportion of permutations for which the maximal component size was greater than m and was then normalized by K. In this way, the NBS attempts to utilize the presence of any structure exhibited by the connections comprising the effect or contrast of interest to yield greater power than what is possible by independently correcting the p-values computed for each link using a generic procedure to control the FWE (11, 12).

Group subdivision. For a *post hoc* analysis, the participants were divided into two groups based on their vocabulary knowledge after six months of learning (above or below average). We used the vocabulary measure extracted from the standardized B1 test to divide the two groups because some subjects did not take the specialized vocabulary test. Both vocabulary tests correlated strongly with B1 language performance (see Figure 1 and Supplementary Figure S3). To illustrate the distribution of connectivity changes for the two groups, we provide the scatterplot in Supplementary Figure S5. This plot shows the relationship between changes (delta) in L2 scores and changes in connectivity (delta connectivity). Note that in the main statistical analysis, we did not us the delta L2 scores, but the absolute values for both time points. The low and high vocabulary groups are plotted separately for the increasing intra-hemispheric connections and the decreasing inter-hemispheric connections.

Supplementary Figures:

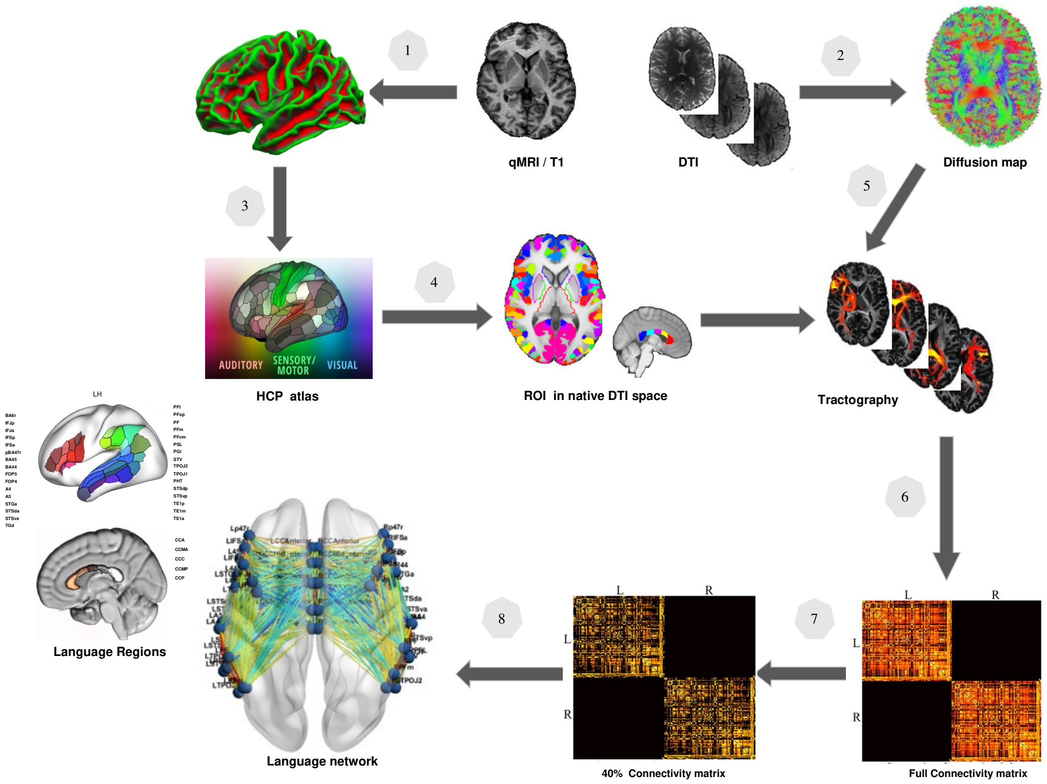

Figure S1: Processing pipeline of structural connectome construction. 1. White matter surface was generated by a segmentation of the anatomical MRI. 2. Preprocessing of the diffusion MRI. 3. Atlas-parcellation of cortical regions in the native brain surface. 4. Registration of the parcellated surface to native diffusion space. 5. Tractography using seed regions in diffusion space. 6. Whole brain network computation. 7. Network thresholding. 8. Extraction of the language network.

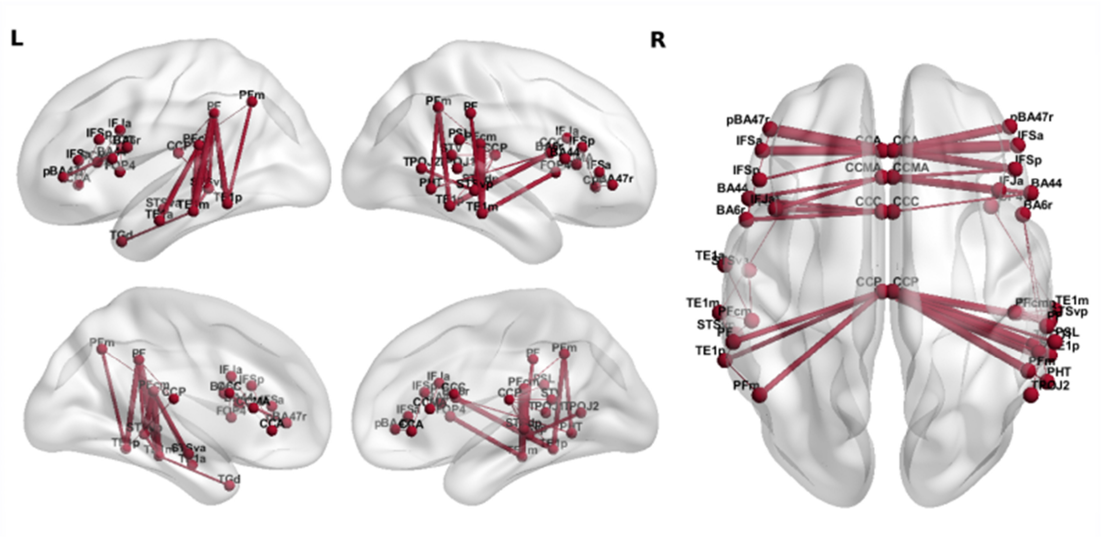

Figure S2: Longitudinal network changes across 3 learning time points. (p<0.05, NBR corrected).

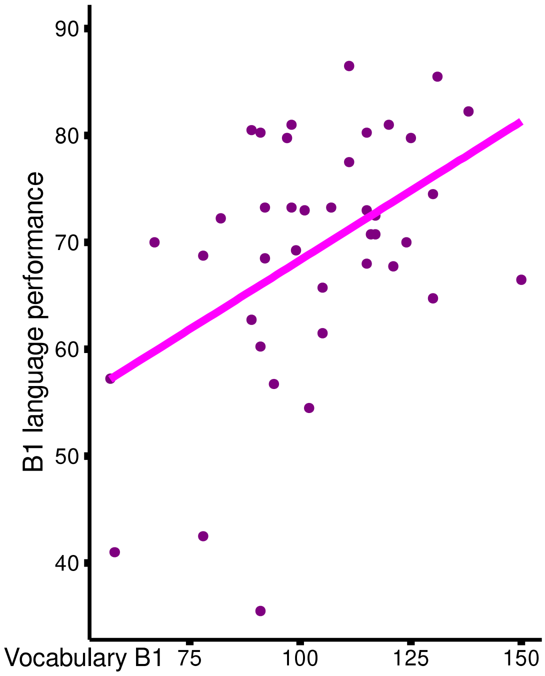

Figure S3: Correlation between L2 vocabularies extracted from the written text in the B1 test and overall language proficiency (B1 test) after six months of L2 learning (r =0.465, p=0.002).

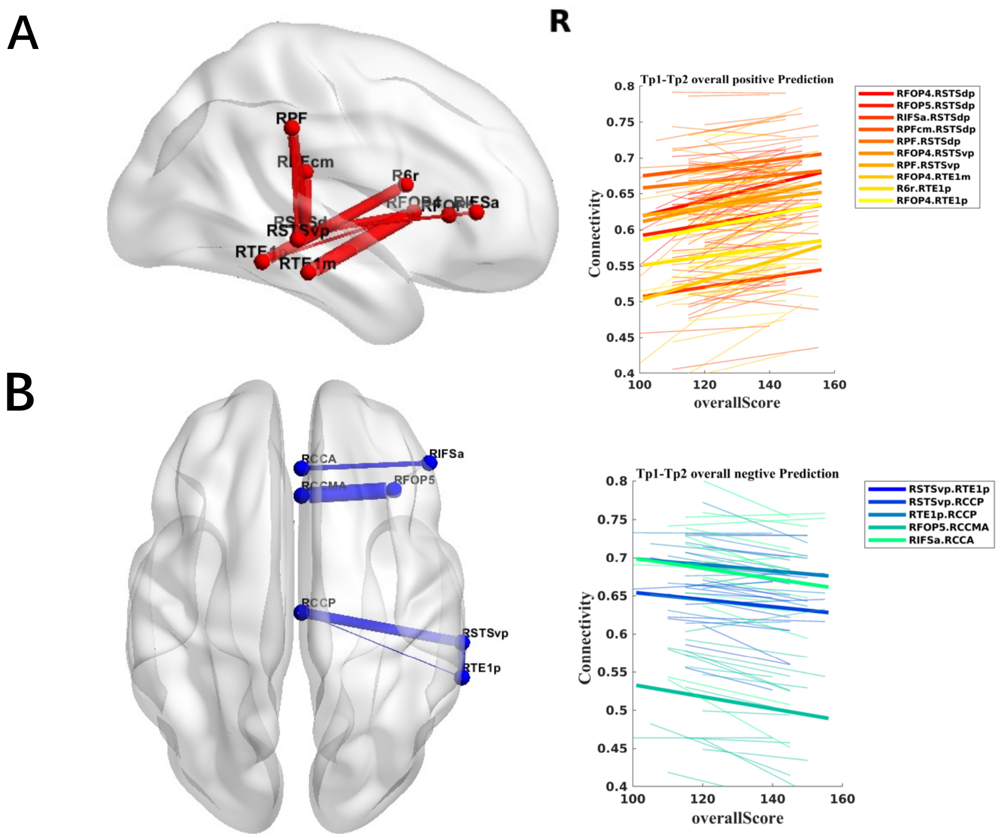

Figure S4: Subgroup with high productive vocabulary scores in the B1 test. Correlation between the changes in connectivity and L2 proficiency from three to six months of L2 learning. Positive (A) and negative (B) correlation between L2 performance and changes in subnetworks. Note that the connection between the right temporal lobe and the frontal lobe follows the right arcuate fascicle as shown in Figure 3 of the main manuscript.

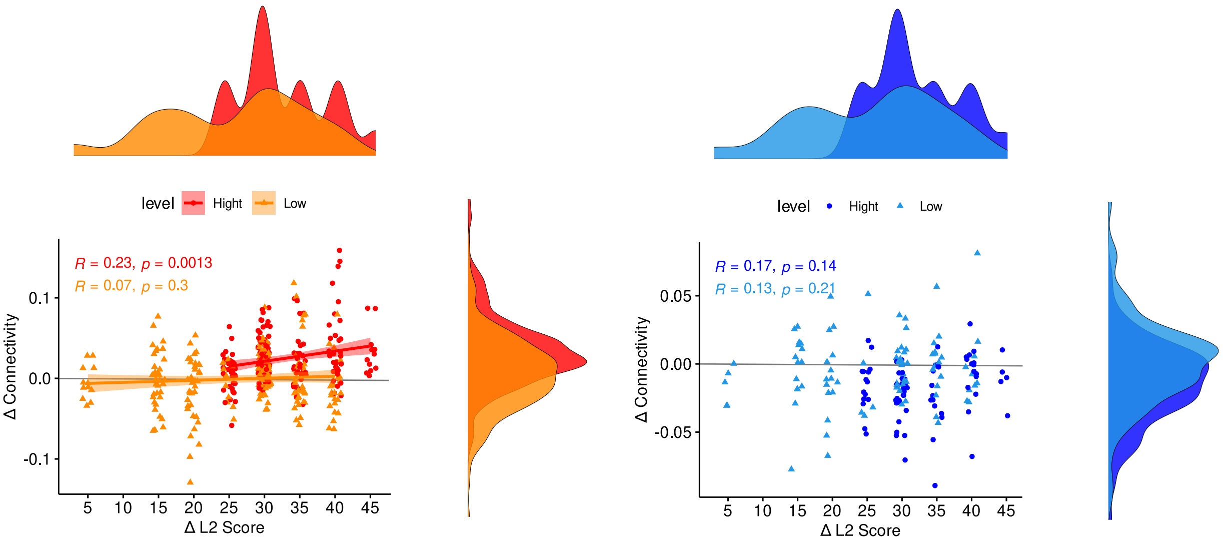

Figure S5: Scatter plot of the groups with a low and high vocabulary score. Left: Increase in L2 score from 3 months to 6 months and the changes in connectivity in the intra-hemispheric cortical network identified in the *post hoc* analysis. The group with the higher vocabulary score shows a correlation (R = 0.23, p=0.0013) and a higher connectivity than the low performance group. The group with the lower vocabulary score showed no change (delta connectivity centered on zero). Right: Decrease in connectivity in the inter-hemispheric network. The group with the higher vocabulary scores showed lower inter-hemispheric connectivity.

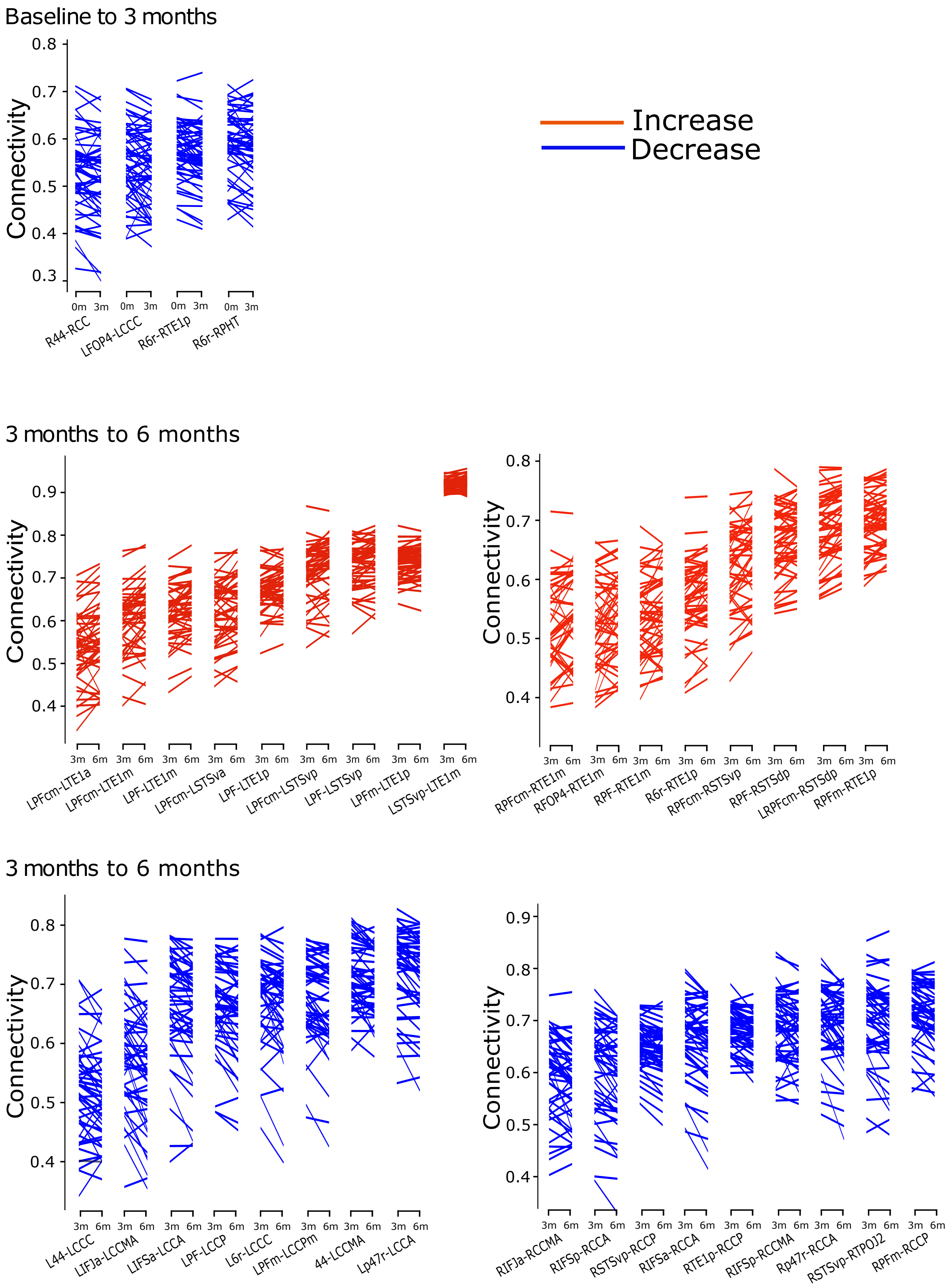

Figure S6: Individual data. Subnetworks with longitudinal increased and decreased connectivity in different L2 learning periods of each participant and each connection within the networks which showed significant changes.

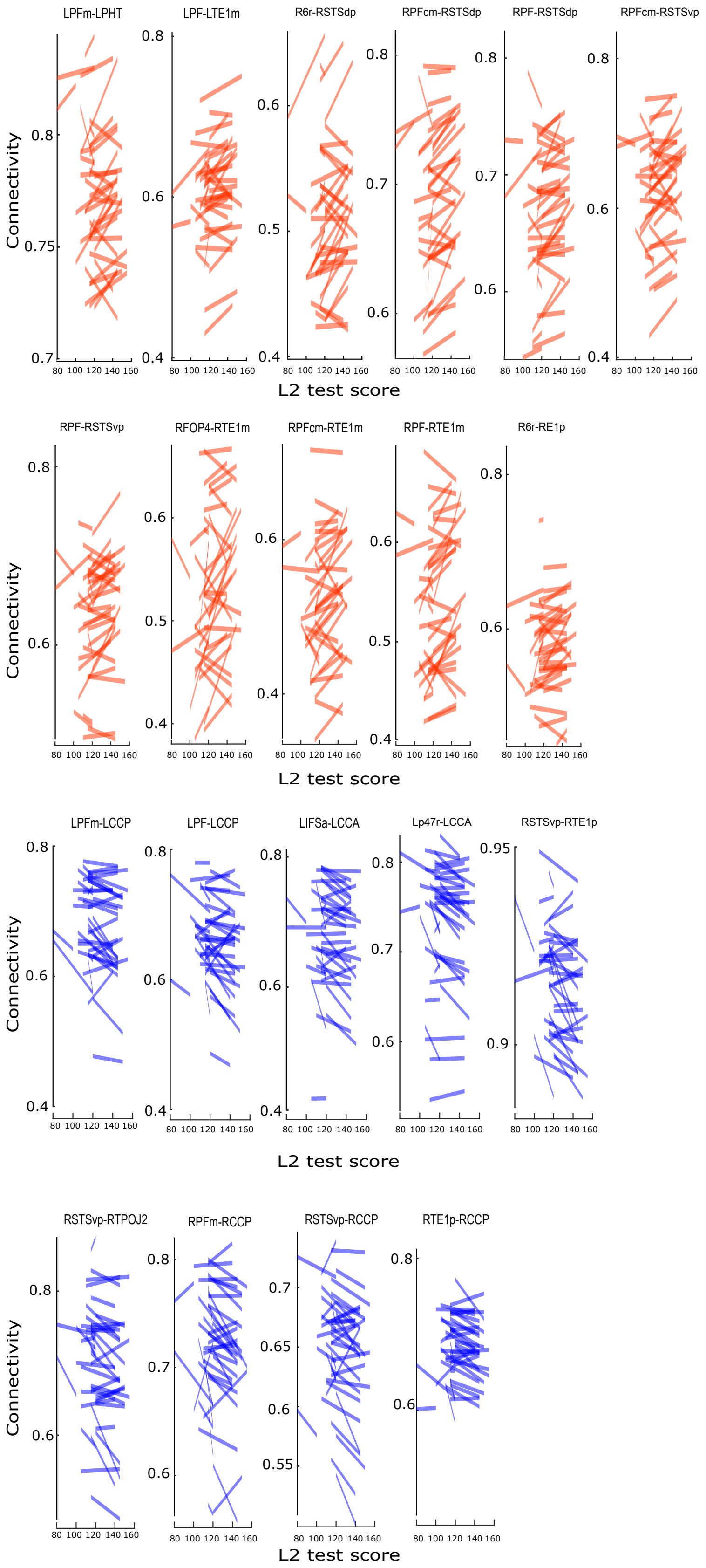

Figure S7: Individual data. Longitudinal changes of the connectivity values in relation to the progress in the language test from 3 months to 6 months of learning (normalized scores).

Supplementary Table 1:

Common Scale	A1	B1
40	0-19.9	
60	20-39.9	
80	40-59.9	0-19.9
100	60-67.4	20-39.9
105	67.5-74.9	-
110	75-82.4	-
115	82.5-89.9	-
120	90-100	40-59.9
140		60-67.4
145		67.5-74.9
150		75-82.4
155		82.5-89.9
160		90-100

Supplementary Table 1: Transformation of the A1 and B1 language test scores to a common scale. The standardized tests require 60% to successfully pass the exam and are less quantitative below this threshold. Therefore, the conversion follows coarser discretization steps of 10 below 60%.

Supplementary Table 2:

HCP Atlas	ROI Name	Area Description	Region
4	BA44	Broca Area 44	IFG
75	BA45	Broca Area 45	IFG
78	BA6r	Broca Area 6 rostral	IFG
79	IFJa	Inferior Frontal Junction anterior	IFG
80	IFJp	Inferior Frontal Junction posterior	IFG
81	IFSp	Inferior Frontal Sulcus posterior	IFG
82	IFSa	Inferior Frontal Sulcus anterior	IFG
108	FOP4	Frontal OPercular area 4	IFG
169	FOP5	Frontal OPercular area 5	IFG
171	pBA47r	posterior Broca Area 47 rostral	IFG
76	47l	Broca Area 47 lateral	IFG
105	PFcm	Area PFcm	IPL
116	PFt	Area PFt	IPL
147	PFop	Area PF opercular	IPL
148	PF	Area PF Complex	IPL

149	PFm	Area PFm Complex	IPL
150	PGi	Area PGi	IPL
25	PSL	PeriSylvian Language area	IPL
140	TPOJ2	Temporo Parieto Occipital Junction 2	IPL
123	STGa	Superior Temporal Gyrus anterior	TL
125	A5	Auditory 5 Complex	TL
128	STSda	Superior Temporal Sulcus dorsal anterior	TL
129	STSdp	Superior Temporal Sulcus dorsal posterior	TL
137	PHT	Area PHT	TL
175	A4	Auditory 4 Complex	TL
176	STSva	Superior Temporal Sulcus ventral anterior	TL
130	STSvp	Superior Temporal Sulcus ventral posterior	TL
131	TGd	Temporal pole dorsal	TL
132	TE1a	Temporal area 1 anterior	TL
177	TE1m	Temporal area 1 middle	TL
133	TE1p	Temporal area 1 posterior	TL
139	TPOJ1	Temporo Parieto Occipital Junction 1	TL
28	STV	Superior Temporal Visual area	TL
--	CCa	Corpus Callosum anterior	aCC
--	CCma	Corpus Callosum middle anterior	aCC
--	CCc	Corpus Callosum central	aCC
--	CCmp	Corpus Callosum middle posterior	pCC
--	CCp	Corpus Callosum posterior	pCC

Supplementary Table 2: Labels for each language region. Inferior Frontal Gyrus: IFG, Temporal Lobe: TL, Inferior Parietal Lobe: IPL, anterior/posterior Corpus Callosum: aCC / pCC.

SI References

1. Council of Europe, *Common European Framework of Reference for Languages: learning, teaching, assessment* (Cambridge University Press, 2001).
2. D. Little, The Common European Framework of Reference for Languages: Content, purpose, origin, reception and impact. *Language Teaching.* **39**, 167–190 (2006).
3. E. Tschirner, *Aligning frameworks of reference in language testing: The ACTFL proficiency guidelines and the common European framework of reference for languages.* (Stauffenburg-Verlag, 2012).
4. E. Tschirner, Examining the validity and reliability of the ITT vocabulary size tests. Research Papers in Assessment. *Univ. Leipzig.* **3** (2021).
5. N. Weiskopf, J. Suckling, G. Williams, M. M. Correia M., B. Inkster, R. Tait, C. Ooi, E. T. Bullmore T., A. Lutti, Quantitative multi-parameter mapping of R1, PD*, MT, and R2* at 3T: A multi-center validation. *Front. Neurosci*. **7**, 95 (2013).
6. T. E. J. Behrens, H. J. Berg, S. Jbabdi, M. F. S. Rushworth, M. W. Woolrich, Probabilistic diffusion tractography with multiple fibre orientations: What can we gain? *Neuroimage.* **34**, 144–155 (2007).
7. A. M. Dale, B. Fischl, M. I. Sereno, Cortical surface-based analysis: I. Segmentation and surface reconstruction. *Neuroimage.* ***9,*** *179-194* (1999).
8. M. F. Glasser, T. S. Coalson, E. C. Robinson, C. D. Hacker, J. Harwell, E. Yacoub, K. Ugurbil, J. Andersson, C. F. Beckmann, M. Jenkinson, S. M. Smith, D. C. Van Essen, A multi-modal parcellation of human cerebral cortex. *Nature.* **536**, 171–178 (2016).
9. C. R. Buchanan, M. E. Bastin, S. J. Ritchie, D. C. Liewald, J. W. Madole, E. M. Tucker-Drob, I. J. Deary, S. R. Cox, The effect of network thresholding and weighting on structural brain networks in the UK Biobank. *Neuroimage.* **211**, 116443 (2020).
10. K. H. Maier-Hein, *et al.*, The challenge of mapping the human connectome based on diffusion tractography. *Nat. Commun.* **8,** 1349 (2017).
11. A. Zalesky, A. Fornito, E. T. Bullmore, Network-based statistic: Identifying differences in brain networks. *Neuroimage.* **53**, 1197–1207 (2010).
12. L. García-Pentón, A. Pérez Fernández, Y. Iturria-Medina, M. Gillon-Dowens, M. Carreiras, Anatomical connectivity changes in the bilingual brain. *Neuroimage.* **84**, 495–504 (2014).
